# Combined medial collateral ligament and posterior oblique ligament reconstruction demonstrates favourable patient‐reported outcomes and medial knee stability in Grade III injuries: A systematic review

**DOI:** 10.1002/ksa.70344

**Published:** 2026-02-19

**Authors:** Ayomide Michael Ade‐Conde, Michelle Cruickshank, Marc Daniel Bouchard, Prushoth Vivekanantha, Meshal Alomari, Darren de SA, Jeffrey Kay

**Affiliations:** ^1^ Royal College of Surgeons in Ireland School of Medicine Dublin Ireland; ^2^ Michael G. DeGroote School of Medicine McMaster University Hamilton Ontario Canada; ^3^ Division of Orthopaedic Surgery McMaster University Hamilton Ontario Canada

**Keywords:** anatomic reconstruction, clinical outcomes, medial collateral ligament, posterior oblique ligament, valgus stability

## Abstract

**Purpose:**

To assess clinical and functional outcomes of combined medial collateral ligament and posterior oblique ligament (MCL–POL) reconstruction in isolated and multiligamentous Grade III medial knee injuries.

**Methods:**

A systematic review was conducted according to PRISMA and Cochrane guidelines. MEDLINE, EMBASE and CENTRAL were searched to 13 August 2025. Studies were eligible if they included patients undergoing combined MCL–POL reconstruction for acute or chronic medial knee instability and reported clinical, functional or radiographic outcomes. Studies involving MCL or POL repair, advancement or non‐reconstructive procedures were excluded. Data on patient characteristics, surgical technique, patient‐reported outcome measures (PROMs), valgus stability, return to sport and complications were extracted.

**Results:**

Twelve studies met the inclusion criteria, involving 350 patients (61.7% female). Mean age was 33.1 ± 4.4 years (range: 27.4–45.6), with mean follow‐up of 41.5 ± 13.5 months (range: 12–86). Mean postoperative Lysholm, subjective International Knee Documentation Committee and Tegner scores were 86.0 (range: 74.8–93.4), 76.3 (range: 66.4–87.8) and 4.9 (range: 3.5–7), respectively, with post‐operative values exceeding established minimal clinically important difference (MCID) thresholds where applicable. Return to recreational or high‐level sport ranged from 64% to 91%. Valgus opening on stress radiographs decreased from 7.5 to 1.2 mm, and 88% of knees were graded as ‘normal’ or ‘nearly normal’ on the IKDC objective scale. The overall complication rate in 327 patients was 20.2%, most commonly arthrofibrosis (5.8%) and superficial infection (4.6%). MCL–POL graft failure was uncommon (1.2%).

**Conclusions:**

This systematic review suggests that MCL–POL reconstruction for Grade III MCL injuries in isolated and multiligamentous injury patterns may provide reliable short to mid‐term clinical and functional outcomes, with greater medial stability than previously reported and acceptable safety profile. However, a lack of standardization and consensus in surgical technique remains, and future prospective studies are needed to determine the comparative efficacy of different techniques.

**Level of Evidence:**

Level IV.

**Abbreviations**
AC ATT: anterior tibial tendon
IFS: interference screw
IKDC: International Knee Documentation Committee
KOOS: Knee Injury and Osteoarthritis Outcome Score
MCID: minimal clinically important difference
MCL: medial collateral ligament
MCL–POL: combined medial collateral ligament and posterior oblique ligament
MINORS: Methodological Index for Non‐Randomized Studies
POL: posterior oblique ligament
PROM: patient‐reported outcome measure
PT: patellar tendon
ROM: range of motion
ST: semitendinosus

## INTRODUCTION

The medial collateral ligament (MCL) and posterior oblique ligament (POL) are well described as critical structures in the posteromedial corner of the knee for resisting valgus and rotatory forces [3, 11, 20, 26, 33, 58]. Grade III medial knee injuries have been shown to involve disruption of both structures in most cases and are often associated with concomitant cruciate ligament injuries [21, 41, 50]. Inadequate restoration of the posteromedial corner can result in persistent laxity, compromised cruciate graft integrity and suboptimal functional outcomes [7, 8]. While isolated MCL injuries often heal without surgery in Grade I and II injuries [15, 27], complete disruption of both the MCL and POL with valgus instability in extension in Grade III is generally considered an indication for operative management [49]. Reconstruction, rather than repair, has become the preferred approach due to more reliable restoration of stability and lower failure rates [40, 49].

Combined MCL and POL (MCL–POL) anatomic reconstruction has gained interest over the past decade as a means to replicate native ligament orientation and tensioning in both isolated and multiligamentous Grade III injuries. Although acute injuries are often managed in the setting of concomitant ligamentous trauma and chronic injuries present with persistent instability following failed non‐operative management, reconstructive principles aimed at restoring valgus and rotatory stability are similar across injury timing in severe cases [32]. Early clinical series in Grade III injuries have demonstrated promising stability, functional outcomes and return‐to‐sport rates with double‐bundle MCL–POL reconstruction techniques [29, 31, 36, 52]. In 2021, a systematic review analyzed these outcomes across six case series and concluded that MCL–POL reconstruction produced satisfactory clinical and functional outcomes [12]. However, the study was limited by a small sample size, low evidence level studies and short follow‐up times. Since then, additional clinical series and technical modifications have been published, supporting the need for an updated synthesis of outcomes and current surgical strategies for MCL–POL reconstruction [13, 28, 37, 45, 48].

The purpose of this systematic review was to provide an evaluation of the available evidence on clinical and functional outcomes of MCL–POL reconstruction for Grade III medial knee instability across isolated and multiligamentous injury patterns. It was hypothesized that this procedure is associated with reliable restoration of stability, satisfactory patient‐reported outcome measures (PROMs) and low complication rates.

## METHODS

### Search strategy

This study was conducted in accordance with Preferred Reporting Items for Systematic Reviews and Meta‐Analysis (PRISMA) and Cochrane handbook guidelines [17, 39]. MEDLINE, EMBASE and CENTRAL databases were searched from inception to 13 August 2025. The search strategy included terms ‘medial collateral’, ‘posterior oblique’ and ‘reconstruction’ (Supporting Information S1: Table S1). The inclusion criteria for this review were (1) studies including patients undergoing simultaneous reconstruction of both the MCL and POL to treat acute or chronic Grade III MCL and/or associated multiligamentous injuries; (2) studies with human participants; (3) studies reporting clinical, functional or radiographic outcomes and (4) studies published in the English language. Grade III injury was defined according to each study's criteria, typically indicating complete disruption of the medial ligament complex with valgus instability in full extension on examination, implying combined disruption of the superficial MCL and POL. Exclusion criteria were (1) studies that assessed POL procedures other than reconstruction (e.g., repair, advancement); (2) editorials, commentaries, case reports, systematic reviews and meta‐ analyses, and Level V evidence; (3) studies involving fewer than five patients and (4) patients who had prior knee surgeries on the same side.

### Screening

The titles and abstracts of retrieved studies were screened by two independent authors in duplicate (A.M.A. and M.C.) in the Covidence online software (Veritas Health Innovation) [10]. Conflicts at this stage were moved on to full‐text review to prevent premature exclusion. Full‐text article screen was also performed in duplicate by the same two authors, with any disagreements resolved by mutual discussion or by consulting a third senior author (M.D.B.) when necessary. Additionally, all reference lists from the included studies were reviewed to verify that no other relevant articles that met the inclusion criteria for this review were omitted.

### Quality assessment

Two authors (A.M.A. and M.C.) assessed the quality of all included studies using the Methodological Index for Non‐Randomized Studies (MINORS) score [51]. For comparative studies, the MINORS score ranges from 0 to 24, where ≥20 is considered high quality, 16–19 is considered good, 11–15 fair, 7–10 low and 0–6 very low quality based on thresholds from a previous systematic review [51]. For non‐comparative studies, the scale ranges from 0 to 16, where ≥13 is considered high quality, 8–12 fair, 5–7 low and 0–4 very low quality [9].

### Data abstraction

An input form in Google Sheets (Google LLC), designed a priori, was used to abstract data from included articles [10]. Two authors (A.M.A. and M.C.) carried out the data abstraction in duplicate, with disagreements resolved by consulting the senior author (M.D.B.).

The study and patient characteristics extracted included the level of evidence, year of publication, number of knees, sex, age, follow‐up duration, time from injury to surgery, injury chronicity (acute vs. chronic), origin of injury and injury type (including associated ligaments and dislocations). Acute and chronic injury status was classified according to definitions used in the included studies, acknowledging variability in how injury chronicity has been defined across the literature. The level of evidence was categorized using the guidelines set by the Oxford Centre for Evidence‐Based Medicine [42].

Surgical details, including the type of graft used, number of bundles, fixation technique, number of femoral and tibial tunnels, concomitant or staged procedures and tensioning protocol for the POL, were abstracted. Rehabilitation protocol details, including use of a brace (yes/no), time to partial weight‐bearing, time to range of motion (ROM) and time to passive or active exercise, were abstracted.

When reported, clinical and functional outcomes abstracted included Lysholm score [54], objective and subjective International Knee Documentation Committee (IKDC) scores [24], Tegner Activity Scale [54], medial joint space and laxity, return to sports and activity, ROM and complication rates. Lysholm scores were categorized as excellent (95–100), good (84–94), fair (65–83) and poor (≤64) [54]. IKDC objective score grades valgus instability according to medial joint space opening relative to the healthy contralateral side on valgus stress test at 20° flexion (Grade A [normal], <2 mm; Grade B [nearly normal], 2–5 mm; Grade C [abnormal], 5–10 mm; Grade D [severely abnormal], >10 mm) [24].

### Statistical analysis

Abstracted data were first checked for normality, after which descriptive statistics in the form of medians and interquartile range (IQR) or weighted means and standard deviation (SD) based on sample size were computed for the study characteristics using Google Sheets (Google LLC). Categorical variables were reported as counts and percentages. Due to the heterogeneity in MCL–POL reconstruction technique and indications, it was not possible to conduct a meta‐analysis, and a narrative summary was presented instead.

## RESULTS

### Search results

**Figure 1:**
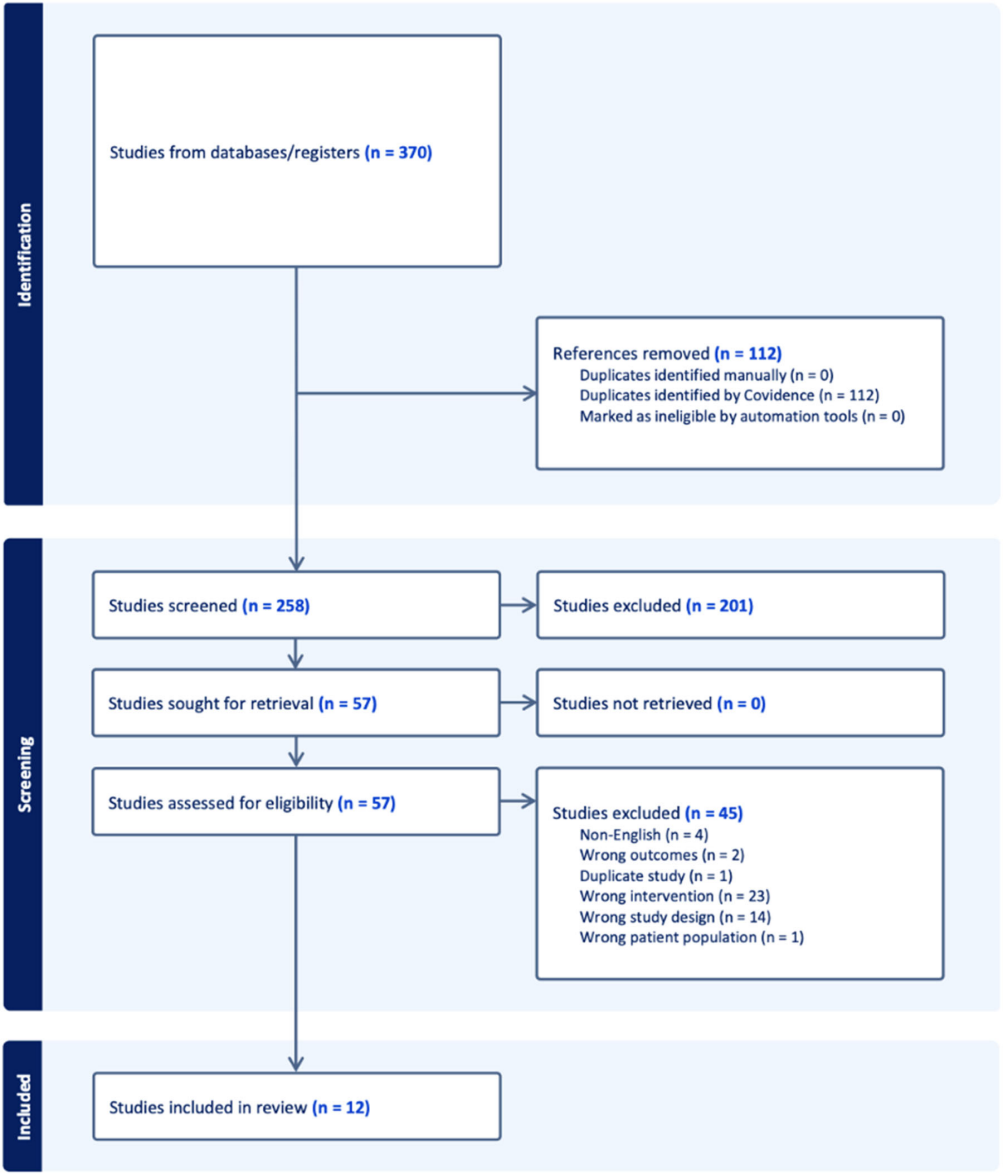
PRISMA flow diagram of included studies. PRISMA, Preferred Reporting Items for Systematic reviews and Meta‐Analyses.

The database search identified 370 articles, with 258 remaining after duplicates were removed (Figure 1). After screening and full‐text review, 12 studies met the inclusion criteria and included Level of Evidence III–IV [1, 14, 22, 25, 29, 31, 34, 36, 44, 52, 53, 59]. Of these studies, three were comparative, and nine were non‐comparative. MINORS scores ranged from 11 to 15 for non‐comparative studies, with a mean of 13.25/16 (high) and 18–24 for comparative studies, with a mean of 19.75/24 (good) (Supporting Information S1: Table S2).

Across all studies, 350 patients who underwent MCL–POL reconstruction for Grade III medial knee instability were included (61.7% female). The weighted mean age was 33.1 ± 4.4 years (range: 27.4–45.6) (Table 1). Across all studies reporting injury timing, 78% of patients were classified as chronic and 22% as acute. The mean post‐operative follow‐up was 41.5 ± 13.5 months (range: 12–86). Isolated Grade III injuries accounted for 14.8% of cases, with the remainder associated with ligamentous knee injuries. The origin of injury was sports‐related in 46.6% of reported cases and road traffic accidents in 38.5% of reported cases.

**Table 1:** Summary of included studies and overall patient demographics.

Author (years)	Study design	Level of evidence	Patients, *n*	Age, y (mean ± SD)	Females, *n* (%)	Time to surgery (mo.)	Acute/chronic	Follow‐up, mo., (mean ± SD)	Origin or mechanism of injury	Injuries, *n*, (%)	Loss to follow‐up, *n* (%)
Abulsoud (2021) [1]	RCS	IV	19	29.63 ± 7.5	4 (21%)	11.2 ± 3	0 (0%)/19 (100%)	NR	NR	Isolated: 5 (26%) ACL + MCL + medial knee structures:10 (53%) PCL + MCL + medial knee structures: 4 (21%)	2 (10.5%)
Denis‐Aubrée (2024) [14]	RCS	IV	26	33.42 ± 11.24	20 (77%)	NR	17 (67%)/9 (33%)	27.4 (range: 12–40)	Sport: 16 (61%) Road accident: 10 (39%)	KD‐I M: 10 (38%); KD‐I PCL: 6 (23%); KD‐I ACL: 4 (15%) KD‐III M: 16 (61%) KD‐IV: (0%) 10 (38%) dislocations (100% KD‐III)	2 (7.7%)
Haroun (2022) [22]	RCS	IV	14	36.6 (range: 23–60)	3 (21%)	1.61	6 (42.9%)/8 (57.1%)	29.5 (range: 24–36)	Road accident: 8 (57%) Work related: 3 (21%)	Isolated: 2 (14%) ACL + medial knee structures: 6 (43%) ACL + PCL + medial knee structures: 3 (21%) PCL + medial knee structures: 2 (14%) PCL avulsion + medial knee structures: 1 (7%)	0
Helito (2022) [25]	RC	III	37	29.8 ± 8.8	8 (21.6%)	6.5 ± 4	0 (0%)/37 (100%)	38.3 ± 7.4	Road accident: 22 (60%) Soccer: 10 (27%) Martial art: 3 (8%) Horse fall: 2 (5%)	ACL + MCL: 19 (52%) ACL + PCL + MCL: 9 (24%) PCL + MCL: 9 (24%)	NR
Kim (2008) [29]	RCS	IV	24	36.3 (range: 7–54)	3 (12.5%)	NR	0 (0%)/24 (100%)	NR	Pedestrian accident: 9 (38%) Sporting activity 9 (38%) Road accident 4 (16%) Motorcycle accident 2 (8%)	Isolated: 6 (25%) ACL + medial knee structures: 12 (50%) PCL + medial knee structures: 6 (25%)	NR
LaPrade (2012) [31]	RCS	IV	28	32.4 (range: 16–56)	9 (32.1%)	NR	8 (28.6%)/ 20 (71.4%)	NR	Valgus contact: 7 (25%) Twist: 5 (18%) Fall: 9 (32%) Road accident: 7 (25%)	Isolated: 2 (7%) ACL + medial knee structures: 8 (29%) PCL + medial knee structures: 9 (32%) ACL + PCL + medial knee structures: 9 (32%)	NR
Lee (2020) [34]	RCS	IV	23	27.4 ± 5.6	9 (39%)	NR	0 (0%)/23 (100%)	77.2 ± 10.8	NR	ACL + medial knee structures: 11 (48%) PCL + medial knee structures: 3 (13%) Previous surgery PCL + PLC + MCL: 3 (13%) Previous surgery ACL + PCL + PLC + MCL: 4 (17%) Failed isolated MCL repair: 2 (9%)	NR
Lind (2009) [36]	RCS	IV	50	34 (range: 14–61)	33 (66%)	NR	0 (0%)/50 (100%)	40 (range: 26–68)	Sports: 41 (82%) Road accident: 7 (14%) Work related: 2 (4%)	MCL isolated: 11 (22%) MCL + ACL: 28 (56%) MCL + ACL + PCL: 3 (6%) MCL + ACL + PCL + PLC: 2 (4%) MCL + PCL + PLC: 1 (2%) MCL + PLC: 1 (2%)	NR
Ortiz (2024) [44]	RC	III	21 Open: 11 Percutaneous: 10	45.6	10 (48.6%)	18.9	3 (14.3%)/ 18 (85.7%)	NR	Sports: 38% NR: 62%	ACL + medial knee structures: (76.2%)	NR
Stannard (2012) [52]	PC	IV	48 ST auto: 27 ATT allo: 21	ST auto: 36.6 ATT allo: 35.3	ST auto: 9/27 (33%) ATT allo: 10/21 (48%)	NR	ST auto: 13 (48%)/14 (52%) ATT allo: 14 (67%)/ 7 (33%)	43 (24–86)	NR	ST auto: Grade 1 (*n* = 1 b); Grade 2 (*n* = 0); Grade 3 (*n* = 3); Grade 4 (*n* = 18); Grade 5 (*n* = 5) ATT allo: Grade 1 (*n* = 1b); Grade 2 (*n* = 0); Grade 3 (*n* = 1); Grade 4 (*n* = 17); Grade 5 (*n* = 2)	42
Tapasvi (2021) [53]	PCS	IV	34	30.6 ± 7.9	11 (32%)	NR	0 (0%)/34 (100%)	49.7 (range: 24–72)	Road accident: 22 (65%) Sports: 8 (23%) Fall: 4 (12%)	Isolated: 2 (6%) ACL + medial knee structures: 25 (74%) ACL + PCL + medial knee structures 6 (17%) PCL + medial knee structures 1 (3%)	0
Xu (2017) [59]	PC	III	26	27.4 ± 4.1	5 (19%)	NR	17 (65%)/9 (35%)	24.3 ± 3.2	NR	Isolated: 26 (100%)	NR
Totals, weighted means/SD	—	—	350	33.1 (27.4–45.6)	134 (38.3%)	—	78 (22%)/272 (78%)	41.5 (12–86)	—	—	—

Abbreviations: ACL, anterior cruciate ligament; KD, Schenck classification of knee dislocation; MCL, medial collateral ligament; mo, months; NR, not reported; PCL, posterior cruciate ligament; PC, prospective cohort; PCS, prospective case series; PLC, posterolateral corner; RC, retrospective cohort; RCS, retrospective case series; SD, standard deviation; y, years.

### Surgical characteristics

All studies except one [1] performed a primary double‐bundle reconstruction with the superficial MCL and POL (Table 2). One study compared open versus percutaneous approaches for MCL–POL reconstruction [44], while another described a minimally invasive single‐bundle technique using a single femoral and tibial tunnel, representing a simplified, non‐anatomic construct [1]. A single femoral tunnel was used in nine studies [1, 22, 25, 29, 34, 36, 44, 52, 59], whereas three studies used two separate femoral tunnels [14, 31, 53]. Concomitant with MCL–POL reconstruction, ACL reconstruction was performed in 35.4% of patients, PCL reconstruction in 9.7% and combined ACL–PCL reconstruction in 8.6%; all procedures were performed simultaneously. For concomitant cruciate reconstruction, three studies [1, 22, 25] reported using contralateral hamstring autografts, while two studies [29, 36] used bone–patellar tendon–bone autografts.

**Table 2:** Surgical characteristics and rehabilitation protocols described by authors.

	Surgical technique									Rehabilitation protocol		
Author (years)	Patients (*n*)	Graft type	Bundle	Femur fixation	Tibial fixation	Femoral tunnel	Tibial tunnel	Tension protocol	Brace (Y/N)	Partial WB time	ROM time	Passive and active exercise time
Abulsoud (2021) [1]	19	ST auto	DB	IFS	Suture	1	1	Knee flexed 30°, axial loading + varus stress during fixation	Y	Day 0	2 wk	2 wk
Denis‐Aubrée (2024) [14]	26	15 CT allo, 7 ATT allo, 1 fibular allo	DB (single slip with anterior slip for MCL, posterior slip for POL)	Ligament staple	IFS	2	2	POL: fixed in neutral rotation and extension; MCL fixed in 30° flexion	Y	Non‐weightbearing for 45 days	NR	Passive ROM from week 1 (limit 90°); active flexion prohibited 6 wk if PCL recon
Haroun (2022) [22]	14	ST auto	DB	IFS	Suture	1	2	POL: fixed at 45° flexion	Y	4 wk (isolated cases), 6 wk (if PCL recon)	4 wk	5 wk
Helito (2022) [25]	37	22 ST auto, 15 allo	DB	NR	NR	1	2	POL: full extension, neutral rotation	Y	Day 0	Immediate ROM if ACL only; delayed 3 wk if PCL involved; meniscal repair limited to 90° first 4 wk	Immediate ROM if ACL only; delayed 3 wk if PCL involved; meniscal repair limited to 90° first 4 wk
Kim (2008) [29]	24	ST auto	DB	SW	Suture	1	2	30° of flexion	Y	2 wk	4/5 wk	4/5 wk
LaPrade (2012) [31]	28	ST allo	DB	IFS	IFS, anchor	2	2	20° of flexion, neutral rotation	Y	6 wk	2 wk	2 wk
Lee (2020) [34]	23	ATT allo	DB	IFS	Suture	1	2	30° of flexion	Y	Day 0	Day 0	2 wk
Lind (2009) [36]	50	ST auto	DB	IFS	IFS	1	2	60° of flexion, neutral rotation	Y	2 wk	3–6 wk	3–6 wk
Ortiz (2024) [44]	21 Open: 11 Percutaneous: 10	PT or AT allo	DB	IFS	IFS	1	2	30° flexion, neutral rotation and varus stress	Y	6 wk non‐WB → progressive loading wk 7–9	0°–90° for 4 wk → gradual increase → full ROM by 6–7 wk	NR
Stannard (2012) [52]	48 ST auto: 27 ATT allo: 21	ST auto: 27, ATT allo: 21	DB	SW	Suture	1	2	30°–40° of flexion, varus stress	Y	0	0	6 wk
Tapasvi (2021) [53]	34	ST auto	DB	IFS	IFS, anchor	2	2	sMCL: fixed 30° flexion + varus; POL fixed full extension + varus	Y	3 wk	NT	NR
Xu (2017) [59]	26	Allo	DB	NR	Suture	1	2	30° of flexion, neutral rotation and varus stress	Y	6 wk	Day 0	6 wk
Total, weighted means/SD	350	Allograft: 160 Autograft: 190	—	—	—	—	—	—	—	—	—	—

Abbreviations: ACL, anterior cruciate ligament; allo, allograft; ATT, anterior tibialis tendon; DB, double‐bundle; IFS, interference screw; MCL, medial collateral ligament; NR, not reported; POL, posterior oblique ligament; PCL, posterior cruciate ligament; ROM, range of motion; sMCL, superficial medial collateral ligament; SD, standard deviation; ST, semitendinosus; SW, staple washer; wk, week; WB, weightbearing.

**Figure 2:**
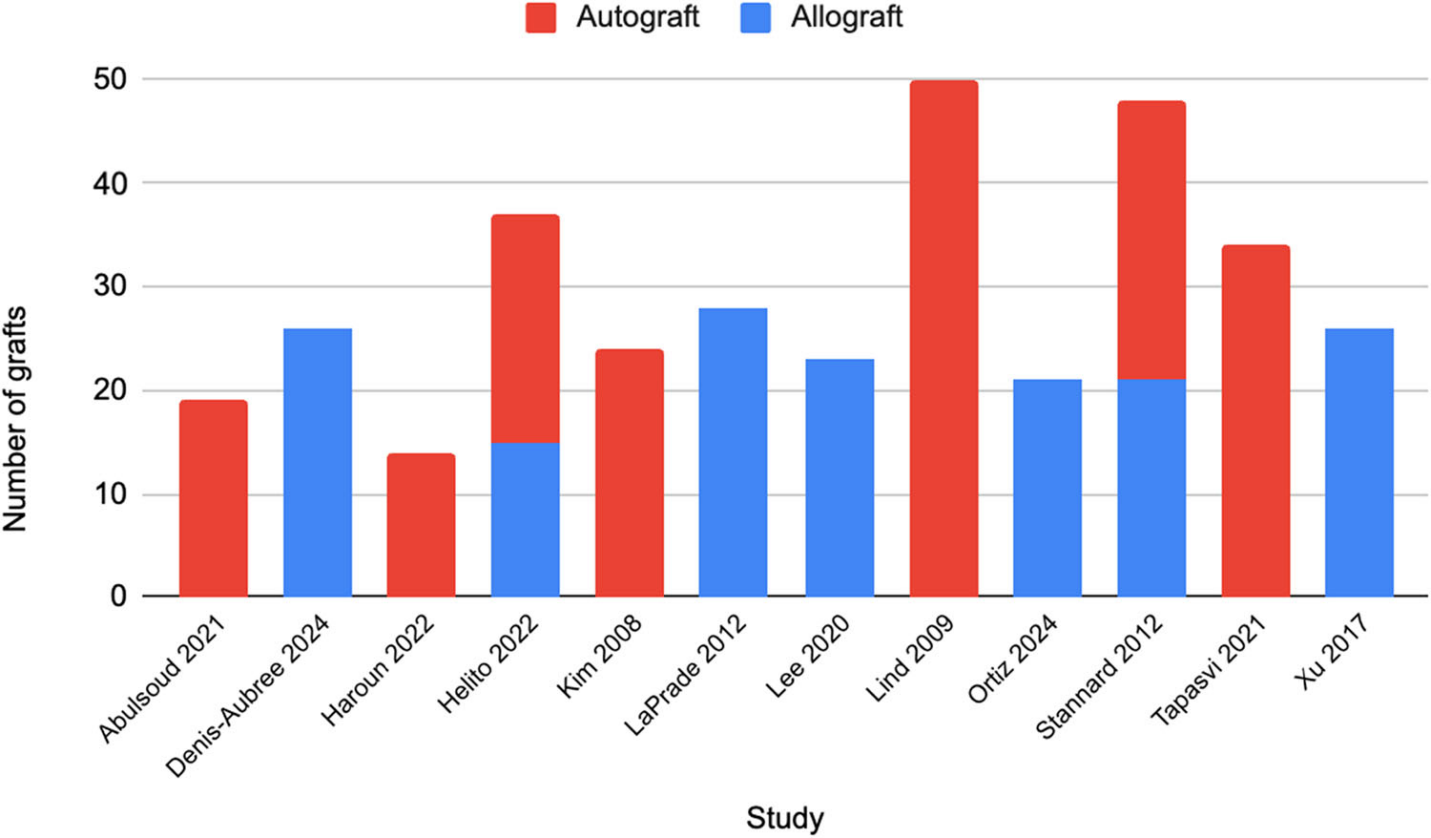
Graft choice distribution across individual studies for MCL–POL reconstruction. MCL–POL, combined medial collateral ligament and posterior oblique ligament.

Among all studies, the MCL–POL reconstruction was performed using an autograft in 190 patients (54.3%) and allograft in 160 patients (45.7%) (Table 2). However, the distribution varied considerably between individual studies (Figure 2). A semitendinosus (ST) autograft was used in seven studies [1, 22, 25, 29, 36, 52, 53]. Among the seven studies using allografts [14, 25, 31, 34, 44, 59], two used an ST allograft [25, 31], four used an anterior tibial tendon (ATT) allograft [14, 34, 44, 52] and one [44] used patellar tendon (PT), and one used an unspecified allograft [59], and another described one‐step anatomic reconstruction with non‐irradiated frozen allografts of the Achilles tendon, fibular tendon, extensor apparatus and PT [14].

The femoral graft‐fixation construct was performed with an interference screw (IFS) in eight studies [1, 22, 31, 34, 36, 44, 53, 59] and a screw washer in two studies [29, 52], and one study used a ligament staple [14] (Table 2). Tibial graft fixation was performed using an IFS in five studies [14, 31, 36, 44, 53] and a suture in six [1, 22, 29, 34, 52, 59].

The tensioning of the POL reconstruction varied in terms of knee flexion. One study tensioned the ligament at 20° [31], seven at 30° [1, 29, 34, 44, 52, 53, 59], one at 60° [36], five in varus stress [1, 44, 52], six in neutral rotation [14, 25, 31, 36, 44, 59] and three in full extension [14, 25, 53].

### Postoperative rehabilitation protocols

All studies incorporated postoperative bracing. Weight‐bearing protocols varied, with four studies [1, 25, 34, 52] permitting immediate partial weight bearing and four studies [14, 31, 44, 59] delaying partial weight bearing until 6 weeks postoperatively. Passive ROM exercises were initiated immediately in four studies [25, 34, 52, 59], while six studies initiated ROM exercises between 2 and 6 weeks postoperatively [1, 25, 29, 31, 36, 44].

### Patient‐reported outcomes

Pre‐operative Lysholm scores were reported in seven studies (*n* = 185), ranging from 27.0 to 85, with a weighted mean of 59.0 (Table 3). Postoperative Lysholm scores were reported at final follow‐up in ten studies (*n* = 272), ranging from 74.8 to 93.4 (Table 3) [1, 14, 22, 25, 29, 34, 44, 52, 53, 59]. Among these, eight reported ‘good’ mean postoperative scores in patients, two reported ‘fair’ scores [14, 25, 54]. The weighted mean was 86.0 postoperatively. Among all studies reporting both pre‐ and postoperative scores, improvements exceeded the MCID of 11.1 points [46]. There were three studies that reported statistically significant improvements from pre‐ to postoperatively (*p* < 0.01) [1, 22, 44].

**Table 3:** Summary of patient‐reported outcomes and medial stability measures following MCL–POL reconstruction.

Author (years)	Patients at FU	Lysholm		IKDC Objective		IKDC subjective		Tegner				
		Pre and post	Passed MCID (Y/N), scale	Pre and post	Passed MCID (Y/N), scale	Pre and post	Passed MCID (Y/N)	Pre and post	Passed MCID (Y/N)/scale	Medial joint opening (mm)	Medial laxity, *n* (%)	ROM flexion
Abulsoud (2021) [1]	19	Pre: 55.7 ± 6.9 Post: 89.4 ± 6.4 (*p* < 0.001)	Y, good	NR	NR	NR	NR	NR	NR		NR	Pre: NR Post: 2 patients had limited flexion (110–120), with full extension
Denis‐Aubrée (2024)[14]	26	Pre: NR Post: 77.78 ± 14.98	NR, fair	Pre: NR Post: Grade A: 20 (77%) Grade B: 6 (22%) Grade C/D: 0 (0%)	NR	Pre: 69.2 ± 17.4	NR	NR	NR	Mean difference in medial femorotibial compartment opening under forced valgus was 0.83 ± 1.26 mm.	Grade I: 16 (84%) Grade II: 3 (16%) Grade: III 0 (0%)	
Haroun (2022)[22]	14	Pre: 27.0 ± 2.9 Post: 87 ± 3.4 (*p* < 0.01)	Y, good	Pre: Grade A: 0 (0%) Grade B: 0 (0%) Grade C: 11 (79%) Grade D: 3 (21%) Post: Grade A: 5 (36%) Grade B: 9 (64%) Grade C: 0 (0%) Grade D: 0 (0%) (*p* < 0.01)	NR	Pre: 46.8 ± 8.3 Post: 71.7 ± 3.68 (*p* < 0.01)	Y	Pre: NR Post: 3.79	NR	NR	The medial laxity graded by IKDC score improved significantly (*p* < 0.01) so that 36% (five patients) had Grade A medial laxity, and 64% (nine patients) had grade B medial laxity at final follow‐up.	NR
Helito (2022)[25]	37	Pre: NR Post: 78.6 ± 16	NR, fair	Pre: NR Post: Posterior: Grade A: 19 (51%) Grade B: 15 (27%) Grade C: 2 (5%) Grade D 1 (3%), Anterior: Grade: A 9 (24%) Grade B: 25 (68%) Grade C: 2 (5%) Grade: D 1 (3%). Medial comp: Grade A: 13 (35%) Grade B: 21 (57%) Grade C: 2 (5%) Grade D: 1 (3%)	NR	Pre: NR Post: 75.8 ± 16.5	NR	NR	NR	NR		Pre: NR Post: Extension loss (mean ± SD): 0.3° ± 1.1° Extension loss >5° (number of patients): 2 patients (5.4%) Flexion loss (mean ± SD): 3.4° ± 4.6° Flexion loss >10° (number of patients): 7 patients (19%)
Kim (2008)[29]	24	Pre: NR Post: 91.9 (80–100)	NR, good	Pre: NR Post: Grade A: 12 (50%) Grade B: 10 (42%) Grade C: 2 (8%) Grade D: 0 (0%)	NR	NR	NR	NR	NR	Pre: 7.8 (range: 5–12) Post: 1.1 (range: 0–5)	NR	Pre: NR Post: Range of movement was normal (lack of extension or flexion of less than 5°) in 19 patients and nearly normal (lack of extension or flexion of less than 10°) in five. No patient had a lack of extension or flexion of more than 10°
LaPrade (2012)[31]	28	NR	NR	NR	NR	Pre: 43.5 (range: 14–65) Post: 76.2 (range: 54–88)	Y	NR	NR	Pre: 6.2 (range: 3.5–14) Post: 1.3 (range: –1 to 2)	NR	NR
Lee (2020)[34]	23	Pre: 49.7 ± 10.2 Post: 93.4 ± 12.4	NR, good	NR	NR	NR	NR	Pre: 5 (range: 4–7) Post: 7 (range: 4–10)	Y, NR	Pre: 8.5 ± 1.6 Post: 1.2 ± 0.7	Valgus laxity at 30° flexion (Grades 0/I/II/III): 16/6/1/0	NR
Lind (2009)[36]	50	NR	NR	Pre: Grade A: 0 (0%) Grade B: 2 (4%) Grade C: 40 (80%) Grade D: 8 (16%) Post: Grade A: 8 (16%) Grade B: 36 (72%) Grade C: 6 (12%) Grade D: 0 (0%)	NR	NR	NR	Pre: NR Post: 4.4 ± 1.8	NR	NR	NR	NR
Ortiz (2024)[44]	21 Open: 11 Percutaneous: 10	Pre: Open: 36.0 ± 10.2 Perc: 47.0 ± 10.3 Post: Open: 74.8 ± 11.9 Perc: 84.2 ± 10 Open: *p* < 0.003 Perc: *p* < 0.001	Open: Y, fair Perc: Y, good	NR	NR	Pre: Open: 34.4 ± 7.1, Perc: 47.2 (35.1–49.9) Post: Open: 66.4 ± 9.4, Perc: 77.9 (66.6–84.6) Open: *p* < 0.001 Perc: *p* < 0.003	Open: Y Perc: Y	Pre: Open: 4.6 ± 0.8, Perc: 5.2 ± 1.4 Post: Open: 3.5 ± 0.8, Perc: 4.1 ± 1.3 Open: *p* < 0.001 Perc: *p* < 0.003	Open: Y, NR Perc: Y, NR	Pre: Open 7.3 (ext), 7.8 (20°); Perc 6.9 (ext), 8.1 (20°) Post: Open 0.3 (ext), 0.4 (20°); Perc 0.2 (ext), 0.4 (20°) (*p* < 0.001)	NR	
Stannard (2012)[52]	48 ST auto: 27 ATT allo: 21	Pre: ST auto: 85 ATT allo: 85 Post: ST auto: 87 ATT allo: 87	NR, good	Pre: NR Post: ST auto: Grade A: 8 (30%) Grade B: 13 (48%) C: 3 (11%) Grade D: 1 (4%) ATT allo: Grade A: 5 (36%) Grade B: 7 (43%) C: 2 (14%) Grade D: 1 (7%)	NR	NR	NR	NR	NR	NR		NR
Tapasvi (2021)[53]	34	Pre: 61.9 ± 11.2 Post: 88.1 ± 7.7	NR, good	Pre: NR Post: Grade A: 11 (32%) Grade B: 21 (62%) C: 2 (6%) Grade D: 0 (0%)	NR	Pre: 58 ± 8.3 Post 78.2 ± 9.5	Y	NR	NR	Pre: 7.5 ± 2.5 Post: 1.2 ± 0.7	Looser: 14 Tighter: 20	NR
Xu (2017)[59]	26	Pre: 49.4 ± 5.3 Post: 90.3 ± 4.5	NR, good	NR	NR	Pre: 47.8 ± 5.1 Post: 87.8 ± 3.6	Y	Pre: 1.6 ± 0.5 Post: 5.7 ± 0.8	NR	NR	NR	NR

Abbreviations: ATT allo, anterior tibial tendon allograft; FU, follow‐up; IKDC, International Knee Documentation Committee (0–100 with higher score being better); Lysholm, Lysholm Knee Scoring Scale (0–100 with higher score being better); MCID, minimal clinically important difference; MCL–POL, combined medial collateral ligament and posterior oblique ligament; NR, not reported; Pre, pre‐intervention; Post, post‐intervention; PT allo, patellar tendon allograft; ROM, range of motion; SD, standard deviation; ST auto, semitendinosus autograft; Tegner, Tegner Activity Scale (0–10 with higher score being better).

Pre‐operative IKDC‐Subjective scores were reported in six studies (*n* = 149), ranging from 34.4 to 69.2 with a weighted mean of 51.9 (Table 3). Postoperative IKDC‐Subjective scores were reported at final follow‐up in seven studies [14, 22, 25, 31, 44, 53, 59], ranging from 66.4 to 87.8, with a weighted mean of 77.2 among 186 patients (Table 3). Among all studies reporting both pre‐ and postoperative scores, improvements exceeded the established MCID of 9.9 [46].

Tegner activity was reported in five studies [22, 34, 36, 44, 59], ranging from 3.5 to 7, with a weighted postoperative mean of 4.9 among 134 patients (Table 3). One study reported a mean reduction of 1.1 from pre‐injury baseline at 12‐month follow‐up [44].

Several comparative analyses were identified in the literature. One study found that IKDC subjective were better in Scheneck classification Grade KD‐I‐M than KD‐III‐M, but no difference in IKDC, Knee Injury and Osteoarthritis Outcome Score (KOOS) and Lysholm between acute and chronic MCL–POL reconstruction [14]. Another found no statistically significant difference in IKDC and Lysholm between MCL–POL reconstruction and MCL advancement [25]. One study reported no significant difference in IKDC‐Subjective or Lysholm scores between patients who had more or less laxity of the MCL than the contralateral knee after reconstruction [53]. Another reported significant improvement in PROMs with both open and percutaneous approaches for MCL–POL reconstruction [44].

### Valgus stability

Valgus opening of the medial compartment was reported in seven studies (*n* = 233) using the IKDC objective scale (Table 3) [14, 22, 25, 29, 36, 52, 53]. Among these, 82 (35.2%) were Graded A and 123 (52.8%) were Graded B, meaning 88% achieved ‘normal’ or ‘nearly normal stability’, while 27 (11.6%) were ‘abnormal’ Graded C and 2 (0.9%) were ‘severely abnormal’ Graded D.

Valgus stress radiographs were used in six studies to assess the extent of medial joint opening [14, 29, 31, 34, 44, 53]. Across these, medial joint space gapping ranged from 0.4 to 1.3 mm, with a weighted mean of 1.0 mm among 156 patients. One study reported a significant decrease in joint space following both open and percutaneous MCL–POL reconstruction (*p* < 0.001) [44].

One study reported significant improvement in anteromedial rotatory stability, with no patients demonstrating residual instability on postoperative anteromedial rotatory drawer (Slocum) testing [22]. No included studies reported formal assessment of posteromedial drawer laxity. Another study reported anteroposterior laxity improved significantly (*p* < 0.001) from a mean of 6.12 mm (range: 2.2–9.1 mm, SD = 1.50) pre‐operatively, to 1.27 mm (range: 0.3–2.6 mm, SD = 0.61) postoperatively [53].

### Return to sport and activity

The return‐to‐sport rate was reported in two studies (Table 3) [34, 36]. In one study, 50 patients (12%) returned to high‐level sports, and 44 (88%) returned to recreational sports after 12 months of postoperative follow‐up [36]. Another study reported that 21 of 23 patients (91.3%) were able to engage in various sports activities, and 15 (65.2%) continued the same level of sports activities after 6.4 years of follow‐up [34].

One study reported nine patients (64.3%) returned at a pre‐injury Tegner activity score level at 2‐year follow‐up [22]. One study reported a KOOS of 81.93 ± 18.06 for activities of daily living and 50.82 ± 28.72 for sports domains at a 12‐month follow‐up [14]. On the other hand, one study evaluating their patients' rate of return to full activity found that of the 71 patients, 35 (49%) returned to their prior level of functioning and 34 (48%) to a decreased level of functioning [52].

### Range of motion

ROM was reported in three studies [1, 25, 29]. One found a mean extension loss of 0.3° with two patients (5.4%) losing more than 5°, and a mean flexion loss of 3.4° with seven (18.9%) patients losing more than 10° [25]. Another study reported five patients (20.8%) losing more than 5°, but none lost more than 10° [29]. One study reported that the MCL–POL advancement technique showed greater loss of ROM for flexion than MCL–POL reconstruction [25].

### Complications

Complication rates were reported in ten studies (*n* = 327), with an overall incidence of 20.2% (*n* = 66). The most frequent were arthrofibrosis (*n* = 19, 5.8%) and superficial wound infection (*n* = 15, 4.6%), none of which required additional surgery (Table 4). In one comparative study, percutaneous MCL–POL reconstruction was associated with a significantly lower rate of arthrofibrosis compared to the open technique (*p* = 0.035) [44]. There were four cases (1.2%) of graft failure reported across two studies at 2‐year follow‐up, with one patient requiring revision surgery [25, 59]. No deep wounds or joint infections were reported across studies.

**Table 4:** Summary of reported complications following MCL–POL reconstruction.

Author (years)	Patients, *n*	Complications								
		Deep infection	Superficial infection	Recurrent instability	Chronic pain	Arthrofibrosis/stiffness	Early osteoarthritis	Ossification	Other	Graft failures
Abulsoud (2021) [1]	19	0	0	0	0	0	0	0	3	0
Denis‐Aubrée (2024) [14]	26	0	0	0	0	5	0	0	0	0
Haroun (2022) [22]	14	0	1	0	0	0	0	0	5	0
Helito (2022) [25]	37	0	2	0	0	4	0	0	1	3
Kim (2008) [29]	24	0	1	0	0	0	0	0	0	0
LaPrade (2012) [31]	28	0	1	0	0	0	0	0	0	0
Lee (2020) [34]	23	NR	NR	NR	NR	NR	NR	NR	NR	NR
Lind (2009) [36]	50	0	0	0	1	1	0	0	3	0
Ortiz (2024) [44]	21 Open: 11 Percutaneous: 10	0	Open: 2 Perc: 1	Open: 2 Perc: 1	0	Open: 4 Perc: 1	Opn: 3 Perc: 1	Open: 1	0	0
Stannard (2012) [52]	48 ST auto: 27 ATT allo: 21	0	6	0	0	4	0	0	0	0
Tapasvi (2021) [53]	34	0	1	0	0	0	0	2	3	0
Xu (2017) [59]	26	0	0	0	0	0	0	2	0	1
Total	327	0	15	3	1	19	4	5	15	4

Abbreviations: Allo, allograft; ATT, anterior tibialis tendon; MCL–POL, combined medial collateral ligament and posterior oblique ligament; NR, not reported; Open, open surgical approach; Perc, percutaneous; ST, semitendinosus.

Minor complications included sensory disturbances (*n* = 6), heterotopic ossification (*n* = 5) and residual instability (*n* = 3). Single cases of cartilage degeneration and persistent pain/inflammation were also described [36]. No thromboembolic events occurred.

## DISCUSSION

The primary findings in this review were that MCL–POL reconstruction for Grade III MCL injuries, in isolation or as part of a multiligamentous knee injury, consistently achieves good short to mid‐term improvements in clinical and functional outcomes. All studies surpassed their respective Lysholm and IKDC‐Subjective MCID thresholds. Objective medial stability also improved markedly, with most knees achieving normal or near‐normal valgus stability on stress radiographs where assessed, indicating reliable restoration of medial restraint.

Combined superficial MCL and POL reconstruction is justified by their complementary roles in resisting valgus and rotatory instability in Grade III injuries [7, 8, 11, 50, 58]. Further, in multiligamentous and ACL‐associated injuries, emerging biomechanical evidence has identified medial ligament deficiency as a key contributor to anteromedial rotatory instability [4, 26]. Compared with a 2021 review reporting a mean postoperative medial opening of 3 mm [12], the present review found a lower mean opening of 1.0 mm, suggesting improved stability outcomes in more recent years. Surgical techniques have also evolved, including modifications inspired by the classic Lind reconstruction [45, 48]. Most studies in this review used a triangular construct, while others adopting true anatomic double‐bundle design with two femoral tunnels described by Lind et al. [36]. Although the latter approach has shown biomechanical superiority and satisfactory functional outcomes [14, 53, 56, 61], simplified anatomic triangular reconstructions have been proposed as cost‐saving and technically easier alternatives that still restore near‐normal stability [19, 34, 59]. Meanwhile, several simplified or minimally invasive modifications to reduce tunnel burden and hardware requirements have recently been proposed, though comparative outcome data remains limited [1, 6, 38, 43, 44, 48]. Overall, contemporary multiligamentous MCL–POL reconstruction appears to achieve greater medial stability than previously reported; however, further evidence is needed to determine whether tunnel configuration influences stability metrics.

PROMs were favourable across studies, with nearly 80% of postoperative Lysholm scores classified as ‘good’. This is consistent with prior literature demonstrating reliable functional improvement after MCL–POL reconstruction in multiligamentous knee injuries [12]. Emerging evidence suggests that patient‐ and injury‐specific factors may also influence outcomes. A recent study found that increased age is associated with lower short‐term KOOS scores [14]. However, the influence of age in other multiligamentous injuries remains debated, with mixed findings reported elsewhere [23, 35]. Patients included in the present review were predominantly young adults sustaining sports‐related knee trauma, reflecting the typical epidemiology of high‐grade MCL injuries [47, 55]. Despite the predominance of athletic injuries across studies, evidence on return‐to‐sport after MCL–POL reconstruction in multiligamentous knee injuries remains limited, underscoring the need for further studies to better define expectations and optimize surgical strategies.

Arthrofibrosis was the most frequently reported complication (5.8%), which is relatively lower than the 8.4%–12.1% rates reported in recent reviews of other complex knee ligament surgical procedures [16, 18]. Acute treatment and ROM‐limited rehabilitation programs have been proposed as potential risk factors [16]. Interestingly, one comparative study suggested a lower arthrofibrosis signal with percutaneous approaches relative to open techniques [44]. However, the evidence remains low‐level, with considerable variability in technique details and soft‐tissue management. Superficial infection (4.6%) was the next most common, while graft failure requiring revision was rare. Overall, MCL–POL reconstruction in multiligamentous knee injuries demonstrates an acceptable safety profile, with arthrofibrosis the main but generally manageable complication. This underscores the importance of early motion protocols and careful soft‐tissue handling.

A recent review identified the Achilles tendon allograft as the most commonly used graft for MCL reconstruction, followed by autologous hamstrings and other soft‐tissue allografts [2]. Allografts may reduce donor‐site morbidity and facilitate single‐stage reconstruction in Grade III injuries; their use is often limited by cost and availability [14, 45, 60]. This limitation was reflected in the present review, with fewer than half of patients receiving MCL–POL allografts. Importantly, superficial infection and graft failure rates were rare across studies, consistent with prior literature demonstrating comparable complication rates between autografts and allografts [5]. Although autograft use may be limited by graft availability and remains theoretically disadvantageous to medial knee stability, the low revision rate observed suggests that this did not translate into a clinically meaningful MCL–POL graft failure [30, 57]. Future comparative studies directly evaluating MCL–POL graft choice are needed to better define its impact on long‐term stability and functional recovery.

Several limitations to this review must be acknowledged. The quality of evidence remains low, as all included studies were retrospective and non‐randomized, limiting causal inference and technique comparison. Substantial heterogeneity in surgical technique, including graft type, tunnel orientation, fixation method and the presence of concomitant ACL reconstruction as a potential cofounder, precluded meta‐analysis. In addition, patient variables such as time to surgery, associated injuries, valgus alignment and rehabilitation protocols were inconsistently reported, limiting subgroup analysis and conclusions about postoperative management. PROMs and return‐to‐sport outcomes were variably reported, with few studies reporting pre‐operative scores, limiting assessment of net improvement and recovery trajectories. Lastly, long‐term outcomes were scarce, with most reporting follow‐up limited to 2–4 years, precluding assessment of graft durability, osteoarthritis progression or revision risk. Future prospective studies with standardized reporting of surgical technique and patient variables are needed to optimize construct selection and to better define patient‐specific expectations.

## CONCLUSION

This systematic review suggests that MCL–POL reconstruction for Grade III MCL injuries in isolated and multiligamentous injury patterns may provide reliable short to mid‐term clinical and functional outcomes, with greater medial stability than previously reported and an acceptable safety profile. However, a lack of standardization and consensus in surgical technique remains, and future prospective studies are needed to determine the comparative efficacy of different techniques.

## AUTHOR CONTRIBUTIONS

All authors contributed to the study design, data collection, analysis and manuscript preparation.

## CONFLICT OF INTEREST STATEMENT

Darren de SA has the following disclosures, none of which are related to this publication. He is a board member of the Heron Therapeutics Advisory Board and has served as a consultant for L.E.K. Consulting, Atheneum Partners and Stryker. Additionally, he has participated in the Speakers Bureau for ConMed Linvatec and is a member of the Pendopharm Regional Working Group. The remaining authors declare no conflicts of interest.

## ETHICS STATEMENT

The authors have nothing to report.

## Supporting information

Supplementary Table 1: Search strategy. Supplementary Table 2: Study‐specific quality assessment.

## Data Availability

The data that support the findings of this study are available in the supporting information of this article.
