# Supplementary material for: Combined medial collateral ligament and posterior oblique ligament reconstruction demonstrates favourable patient‐reported outcomes and medial knee stability in Grade III injuries: A systematic review
Source: Knee Surg Sports Traumatol Arthrosc. 2026 Feb 19;34(4):1292–309. doi: 10.1002/ksa.70344 (PMC13037351; doi:10.1002/ksa.70344)
Supplement: Supplementary file 1 — Supplementary Table 1: Search strategy. Supplementary Table 2: Study‐specific quality assessment. [file KSA-34-1292-s001.docx]

**Supplemental Digital Content Table 1** Search Strategy

| MEDLINE, EMBASE and CENTRAL via OVID |
| --- |
| 1. posterior oblique*.ti,ab,kw 2. posteromedial capsule*.ti,ab,kw 3. posteromedial corner*.ti,ab,kw 4. 1 OR 2 OR 3 5. reconstruct*.ti,ab. 6. anatomic reconstruct*.ti,ab. 7. 5 OR 6 8. 4 AND 7 |

**Supplemental Digital Content Table 2** Study-specific quality assessment

| **Criteria** | ***Abulsoud (2021)*** | ***Aubrée (2024)*** | ***Haroun (2022)*** | ***Helito (2022)*** | ***Kim (2008)*** | ***LaPrade (2012)*** | ***Lee (2020)*** | ***Lind (2009)*** | ***Ortiz (2024)*** | ***Stannard (2012)*** | ***Tapasvi (2021)*** | ***Xu (2017)*** |
| --- | --- | --- | --- | --- | --- | --- | --- | --- | --- | --- | --- | --- |
| **1. A clearly stated aim** | **2** | **2** | **2** | **2** | **2** | **2** | **2** | **2** | **2** | **2** | **2** | **2** |
| **2. Inclusion of consecutive patients** | **2** | **2** | **2** | **2** | **2** | **2** | **2** | **2** | **2** | **2** | **2** | **2** |
| **3. Prospective collection of data** | **2** | **0** | **0** | **1** | **0** | **2** | **2** | **0** | **0** | **2** | **2** | **0** |
| **4. Endpoints appropriate to the aim of study** | **2** | **2** | **2** | **2** | **2** | **2** | **2** | **2** | **2** | **2** | **2** | **2** |
| **5. Unbiased assessment of study endpoint** | **2** | **2** | **2** | **2** | **2** | **1** | **1** | **2** | **2** | **1** | **1** | **1** |
| **6. Follow-up period appropriate for the aim of study** | **2** | **2** | **2** | **2** | **2** | **1** | **2** | **2** | **2** | **2** | **2** | **2** |
| **7. Loss to follow-up less than 5%** | **1** | **1** | **2** | **2** | **1** | **2** | **2** | **1** | **2** | **0** | **2** | **2** |
| **8. Prospective calculation of study size** | **0** | **0** | **1** | **1** | **0** | **0** | **2** | **0** | **2** | **0** | **2** | **0** |
|  |  |  |  |  |  |  |  |  |  |  |  |  |
| **9. An adequate control group** | **-** | **2** | **-** | **2** | **-** | **-** | **-** | **-** | **2** | **2** | **-** | **-** |
| **10. Contemporary groups** | **-** | **2** | **-** | **2** | **-** | **-** | **-** | **-** | **1** | **2** | **-** | **-** |
| **11. Baseline equivalence of groups** | **-** | **1** | **-** | **2** | **-** | **-** | **-** | **-** | **2** | **1** | **-** | **-** |
| **12. Adequate statistical analyses** | **-** | **2** | **-** | **2** | **-** | **-** | **-** | **-** | **2** | **2** | **-** | **-** |
| **TOTAL MINORS SCORE** | **13** | **18** | **13** | **22** | **11** | **12** | **15** | **11** | **21** | **18** | **15** | **11** |
| **Maximum possible score** | **16** | **24** | **16** | **24** | **16** | **16** | **16** | **16** | **24** | **24** | **16** | **16** |
